# Engaging clinicians and patients to assess and improve frailty measurement in adults with end stage renal disease

**DOI:** 10.1186/s12882-017-0806-0

**Published:** 2018-01-12

**Authors:** Sarah Van Pilsum Rasmussen, Jonathan Konel, Fatima Warsame, Hao Ying, Brian Buta, Christine Haugen, Elizabeth King, Sandra DiBrito, Ravi Varadhan, Leocadio Rodríguez-Mañas, Jeremy D. Walston, Dorry L. Segev, Mara A. McAdams-DeMarco

**Affiliations:** 1 Department of Surgery, Johns Hopkins University School of Medicine, Baltimore, MD USA; 2 Department of Epidemiology, Johns Hopkins School of Public Health, Baltimore, MD USA; 3 Department of Medicine, Johns Hopkins University School of Medicine, Baltimore, MD USA; 4 Department of Oncology, Sidney Kimmel Comprehensive Cancer Center, Baltimore, MD USA; 5 Hospital Universitario de Getafe, Madrid, Spain; 6 Department of Epidemiology, 615 N. Wolfe St, W6033, Baltimore, MD 21205 USA

**Keywords:** Frailty, Hemodialysis, ESRD

## Abstract

**Background:**

The Fried frailty phenotype, a measure of physiologic reserve defined by 5 components (exhaustion, unintentional weight loss, low physical activity, slow walking speed, and poor grip strength), is associated with poor outcomes among ESRD patients. However, these 5 components may not fully capture physiologic reserve in this population. We aimed to ascertain opinions of ESRD clinicians and patients about the usefulness of the Fried frailty phenotype and interventions to improve frailty in ESRD patients, and to identify novel components to further characterize frailty in ESRD.

**Methods:**

Clinicians who treat adults with ESRD completed a 2-round Delphi study (*n* = 41 and *n* = 36, respectively; response rate = 87%). ESRD patients completed a survey at transplant evaluation (*n* = 460; response rate = 81%). We compared clinician and patient opinions on the constituent components of frailty.

**Results:**

Clinicians were more likely than patients to say that ESRD makes patients frail (97.6% vs. 60.2%). There was consensus among clinicians that exhaustion, low physical activity, slow walking speed, and poor grip strength characterize frailty in ESRD patients; however, 29% of clinicians thought weight loss was not relevant. Patients were less likely than clinicians to say that the 5 Fried frailty components were relevant. Clinicians identified 10 new ESRD-specific potential components including falls (64%), physical decline (61%), and cognitive impairment (39%). Clinicians (83%) and patients (80%) agreed that intradialytic foot-peddlers might make ESRD patients less frail.

**Conclusions:**

There was consensus among clinicians and moderate consensus among patients that frailty is more common in ESRD. Weight loss was not seen as relevant, but new components were identified. These findings are first steps in refining the frailty phenotype and identifying interventions to improve physiologic reserve specific to ESRD patients.

**Electronic supplementary material:**

The online version of this article (10.1186/s12882-017-0806-0) contains supplementary material, which is available to authorized users.

## Background

Frailty is a phenotype of decreased physiologic reserve resulting in a vulnerability to adverse health outcomes upon confrontation by stressors. The phenotype was originally defined and validated in older populations by Fried et al. as the presence of at least 3 of 5 components: exhaustion, unintentional weight loss, low physical activity, slow walking speed, and poor grip strength [1]. Among older adults, frailty prevalence ranges from 7 to 15% and is associated with increased risk of mortality, incident disability, hospitalizations, and falls [1–5]. Among end stage renal disease (ESRD) patients, the prevalence of the Fried frailty phenotype is 5 to 7-fold higher than in community-dwelling older adults [6] and is associated with higher mortality, more hospitalizations, more falls, poor cognitive function, and poor health-related quality of life [6–9]. Among ESRD patients who undergo kidney transplantation (KT), the Fried frailty phenotype is associated with worse health related quality of life and physical function at the time of KT and longer post-KT hospital stay, delayed graft function, early hospital readmission, immunosuppression intolerance, and increased mortality [6, 9–15].

Despite the importance of the Fried frailty phenotype in risk stratification of clinical outcomes in ESRD patients, its predictive ability is moderate (area under the receiver operative curve = 0.70; c statistic = 0.75) [11, 12] suggesting that the phenotype may be missing some valuable information for ESRD patients. Because the Fried frailty phenotype was developed in community-dwelling older adults, some components may not be applicable to ESRD patients, and there may be aspects of physiologic reserve in ESRD patients that are not characterized by this phenotype. Supporting this hypothesis, we have previously found that not all 5 components contribute to the value of the Fried phenotype in predicting mortality risk among KT recipients [16].

A more accurate measure of frailty among ESRD patients will improve risk prediction, aid in clinical decision-making, develop targeted patient counseling, and identify interventions to improve physiologic reserve. Both ESRD patient and clinician opinions are needed to identify which of the 5 Fried components are mechanistically and biologically plausible, and which ESRD-specific components should be added or substituted in the phenotypic description of frailty to improve characterization of physiologic reserve in this population.

The primary goal of this study was to ascertain the opinions of experts who care for patients with ESRD and of ESRD patients pursuing kidney transplantation about the usefulness of the Fried frailty phenotype, and which novel components may further characterize frailty among ESRD patients. Additionally, we sought to elicit the opinions of these clinicians and patients on the effectiveness of and patient interest in interventions to improve frailty in ESRD and transplant patients. The Delphi method [17], an iterative survey design that builds consensus among experts through a series of structured surveys, has been used to elicit opinions on frailty among geriatrics experts [18]. In this study, we used the Delphi method to evaluate clinician consensus on the constituent components of frailty in ESRD and contrast those opinions with those of ESRD patients.

## Methods

### Delphi survey of clinicians

Since typical Delphi panels have ranged in size from 15 to 60 participants [17], we identified 53 attending nephrologists, geriatricians, and transplant surgeons who care for adults with ESRD at Johns Hopkins. Using a two-round Delphi approach, we distributed two anonymous surveys (Additional file 1: Table S1) to clinicians at a single center 1 month apart. Responses were only collected from clinicians who said they treated adults with ESRD. The first survey was distributed to 53 participants, and the second survey was distributed to the 46 clinicians that responded to the first survey. To preserve the anonymity of the surveys, we sent the second survey to all respondents in the first survey regardless of whether they treated patients with ESRD. Participants were recruited over email and the surveys were administered online by Qualtrics (Provo, UT). In the survey, frailty was defined as “a syndrome characterized by a loss of physiologic reserve.” The five Fried frailty components were also listed.

The first round Delphi survey included qualitative and quantitative questions on frailty and interventions for ESRD patients (Additional file 1: Table S1). Clinicians were asked if they would add components to or remove components from the Fried frailty definition (Additional file 2: Table S2) to characterize frailty in adults with ESRD, and if so they were asked to list the components and explain their answers. Novel components were generated from open-ended questions in the first survey and were not suggested by the researchers. Clinicians were also asked their opinions on the efficacy of and patient interest in interventions to improve frailty such as prehabilitation, and intradialytic activities such as foot peddlers. The second round Delphi survey presented participants with results from the first survey to: 1) inform participants of the current status of the group’s opinion, 2) help participants identify responses that they missed or gave low importance in previous rounds, and 3) allow participants to change their opinion [17] (Additional file 3: Table S3).

### Survey of ESRD patients

To elicit patient opinions, we conducted a survey of adults (aged ≥18 years old) with ESRD at the time of evaluation for KT and who had a Fried frailty phenotype measurement (Additional file 4: Table S4). All participants were recruited from Johns Hopkins. Frailty was defined as the presence of three or more of the five Fried frailty components: exhaustion, unintentional weight loss, low physical activity, slow walking speed, and poor grip strength (Additional file 4: Table S4) [1]. Surveys were conducted in person or over the phone and consisted of 10 questions about the five Fried frailty components. They were also asked 4 questions about the potential efficacy of and their interest in prehabilitation prior to KT to improve frailty. ESRD patients who were on hemodialysis at the time of the survey answered three questions about the potential efficacy of and their interest in intradialytic foot peddlers to improve frailty. Before beginning the survey, participants were told that “frailty is a syndrome characterized by a loss of physiologic reserve. People who are frail are unable to bounce back after they get sick or hurt.” Participants’ Fried frailty measurements were ascertained using the Fried measure of frailty (Additional file 2: Table S2) [1].

The Delphi study of clinicians and the survey of patients received human subjects research approval from the Johns Hopkins Medicine Institutional Review Board.

### Statistical analysis

Summary statistics were calculated using Stata 14. Qualitative responses to the first round of the Delphi study were analyzed using NVivo. Chi-squared and Fisher’s exact tests were used to examine differences between patients and clinicians, and by clinician characteristics (specialty, years in practice, and age) and patient characteristics (age, race, sex, time on dialysis, and measured Fried frailty status). To preserve anonymity, we combined the nephrologist and transplant surgeon responses; only 3 transplant surgeons who treated patients with ESRD responded to the survey. As has been previously defined, consensus was defined was >80% agreement, and moderate consensus was defined as 60–80% agreement [17–19].

## Results

### Survey response rates and participant characteristics: Clinicians

The response rate for the first survey was 87% (46 of 53) and the second survey was 87% (40 of 46); only clinicians who responded to the first survey were asked to complete the second survey. Five respondents of the first survey and four respondents of the second survey did not treat adults with ESRD and were dropped from further analysis, resulting in a sample of 41 and 36 clinicians for the first and second surveys, respectively. Of the clinicians who responded to the survey, 61% were transplant surgeons or nephrologists, 39% were geriatricians, 76% were >40 years old, 46% were female, and 83% had been in practice for ≥5 years.

### Survey response rate and participant characteristics: ESRD patients

There were 460 adults with ESRD who responded to the patient survey for a response rate of 80.6%. Surveyed patients were 39% female and 46% white; 63% were <60 years old and 56% were being treated with hemodialysis (Table 1). Only 27 patients (6%) self-identified as frail (mean age = 54.4, standard deviation (SD) = 14.14), while 95 patients (20%) were measured as frail by the Fried phenotype (mean age = 56.5, SD = 11.4); of the 27 patients who self-identified as frail, 25 (93%) were measured as either frail or intermediately frail. All participants had an eGFR of 20 ml/min or less.

**Table 1:** ESRD patient survey population characteristics by measured frailty status

Participant Characteristics	Frail n (%) 95 (20.65)	Not frail n (%) 365 (79.35)	Overall n (%) 460
Sex			
Female	35 (36.8)	143 (19.2)	178 (38.7)
Race			
White	40 (42.1)	173 (47.4)	213 (46.3)
African American	50 (52.6)	159 (43.6)	209 (45.4)
Asian	3 (3.2)	17 (4.7)	20 (4.4)
Native Hawaiian/Pacific Islander	0 (0)	2 (0.6)	2 (0.4)
Other	2 (2.1)	14 (3.8)	16 (3.5)
Age			
< 30	2 (2.1)	19 (5.2)	21 (4.6)
30–39	8 (8.4)	37 (10.1)	45 (9.8)
40–49	16 (16.8)	69 (18.9)	85 (18.5)
50–59	27 (28.4)	113 (40.0)	150 (30.4)
60–69	34 (35.8)	93 (25.5)	127 (27.6)
≥ 70	8 (8.4)	34 (9.3)	42 (9.1)
Dialysis Type			
Hemodialysis	56 (59.0)	196 (55.4)	252 (56.1)
Peritoneal dialysis	12 (12.6)	46 (13.0)	58 (12.9)
Not on dialysis	27 (28.4)	112 (31.6)	139 (31.0)
Time on Dialysis Among Patients on Dialysis (years)			
< 1	14 (29.8)	31 (17.3)	45 (19.9)
1–1.9	21 (44.7)	57 (31.8)	78 (34.5)
2–2.9	7 (14.9)	34 (19.0)	41 (18.1)
3–3.9	1 (2.1)	15 (8.4)	16 (7.1)
4–4.9	3 (6.4)	15 (8.4)	18 (8.0)
≥ 5 years	1 (2.1)	27 (15.1)	28 (12.4)

### Opinions on frailty in adults with ESRD

Ninety-eight percent of clinicians and 62% of ESRD patients felt that adults with ESRD are more likely to be frail than healthy adults (Table 2). There were three Fried frailty components that at least one clinician identified as not relevant to adults with ESRD: weight loss, slowed walking, and weak strength (Table 3). In both surveys, the component most frequently identified as not relevant to adults with ESRD was weight loss (20% in the first and 29% in the second survey); in a free-response question, five clinicians explained that weight fluctuates in this population. Opinions on removing weight loss from the Fried frailty phenotype were associated with specialty such that nephrologists and transplant surgeons were less likely than geriatricians to want to remove weight loss (10% vs. 50%, *p* = 0.02). Patient perceptions of the relevance of the five Fried frailty components differed from those of clinicians; clinicians were more likely to say that weight loss (80% vs. 62%) walking speed (92% vs. 51%), physical activity (100% vs. 67%), weak grip strength (97% vs. 81%), and exhaustion (100% vs. 80%) were relevant to frailty among ESRD patients.

**Table 2:** Clinician and patient opinions on frailty in adults with ESRD and on intradialytic interventions

	% Clinicians in first survey	% Clinicians in second survey	% Patients
Are ESRD patients more likely to be frail than healthy adults?			
More likely	97.6	*	61.8
Less likely	0	*	9.13
About the same	2.4	*	29.0
Relevance of frailty component			
Weight loss	80.0	71.4	61.6
Walking speed	92.5	94.3	51.2
Physical activity	100	*	67.0
Weak Strength	97.5	100	81.2
Exhaustion	100	*	80.0
Opinions on Interventions to improve frailty			
Patients would be interested in foot peddlers	69.2	*	62.5
Foot peddlers would make patients less frail	83.3	*	79.7
Opinions on prehabilitation to improve frailty			
Patients would be interested in prehabilitation	97.1	*	80.2
Prehabilitation would make ESRD patients less frail	97.1	*	84.5
Prehabilitation would help ESRD patients	100	*	93.8

^*^Questions on the relevance of frailty components were only included in the second survey of clinicians if there was not total agreement in the first survey. Questions on interventions, prehabilitation and prevalence of frailty in ESRD patients were not included in the second survey due to consensus and moderate consensus (>60% agreement) reached in the first survey

**Table 3:** Clinician opinions on Fried Frailty components and novel ESRD-specific frailty components

**A. Percentage of clinicians identifying components as not relevant to adults with ESRD in the first and second survey**			
**Component**	**% identifying in 1st survey (n = 41)**	**% identifying in 2nd survey (n = 36)**	**Qualitative explanations for irrelevance (1st survey)**
Unintentional Weight Loss	20.0	28.6	- Weight fluctuates in this population (*n* = 5) - Not relevant to ESRD population (*n* = 2)
Slowed Walking	7.5	6.0	Not relevant to ESRD Population (n = 2)
Weak Strength	2.5	0	Generalized fatigue is more relevant (*n* = 1)
Low Physical Activity	0	*	n/a
Exhaustion	0	*	n/a
**B. Novel ESRD-specific components suggested by clinicians in the first round, and percentage of clinicians wanting to add each component by specialty in the second round**			
**1st survey: components identified**	**2nd survey: % wanting to add component**	**% wanting to add component by specialty**	
		**Geriatricians**	**Nephrologists and Transplant Surgeons**
History of falls	63.9	37.5	85.0
Physical decline	61.1	50.0	70.0
Cognitive Impairment	38.9	18.8	55.0
Nutrition, diet	36.1	25.0	45.0
Albumin	16.7	6.2	25.0
Health care utilization	11.1	12.5	10.0
Metabolic bone disease	5.6	0	10.0
Excess fluid	2.8	0	5.0
Ultrafiltration	2.8	0	5.0

^*^Questions on the relevance of frailty components were only included in the second survey of clinicians if there was not total agreement in the first survey

Among ESRD patients, 91% of participants who both measured and self-identified as frail believed that adults with ESRD are more likely to be frail than healthy adults. However, 58% of participants who measured as frail but self-identified as non-frail believed that adults with ESRD are more likely to be frail (Table 4).

**Table 4:** Characteristics of ESRD patients by measured and self-identified frailty status

Measured as Frail Self-identified as frail N	Yes Yes 12	Yes No 83	No Yes 15	No No 350
Age (median (IQR))	52.9 (44.1–66.1)	59.0 (49.5–65.6)	56.9 (48.8–65.4)	54.5 (46.7–64.3)
Male	58.3%	63.9%	66.7%	60.6%
Race				
White/Caucasian	41.7%	42.2%	46.7%	47.4%
Black/African American	58.3%	51.8%	46.7%	43.4%
Other	0%	6.0%	6.7%	9.2%
Are ESRD patients more likely to be frail than healthy adults?				
More likely	90.9%	58.2%	73.3%	61.2%
Less likely	0%	3.8%	0%	11.2%
About the same	9.1%	37.9%	26.7%	27.6%

### Novel ESRD-specific frailty components

Clinicians identified ten novel components that potentially characterize frailty in adults with ESRD (Table 3). In the second survey, there was moderate consensus that history of falls (64%) and physical decline (61%) should be added; additionally, 39% of clinicians identified cognitive impairment as a novel component. Nephrologists and transplant surgeons were more likely to want to include cognitive impairment and history of falls as ESRD-specific frailty components than geriatricians (cognitive impairment: 55% vs. 19%, *p* = 0.04; falls: 85% vs. 38%, *p* = 0.005).

### Intradialytic activities to improve frailty among adults with ESRD

Among clinicians there was consensus (83% agreement) and among ESRD patients who were on hemodialysis there was moderate consensus (80% agreement) that intradialytic foot peddlers will make ESRD patients on dialysis less frail (Table 2). However, there was only moderate consensus among both clinicians (69% agreement) and patients (62% agreement) that ESRD patients would be interested in using foot peddlers while undergoing hemodialysis.

### Prehabilitation to improve frailty among adults undergoing kidney transplant

There was consensus among both clinicians (97% agreement) and patients (94% agreement) that pre-KT prehabilitation will help patients undergoing transplantation (Table 2) and that prehabilitation would make ESRD patients less frail (100% vs. 84%, Table 2). Among clinicians there was consensus (97% agreement) and among patients there was moderate consensus (80% agreement) that patients would be interested in pre-KT prehabilitation (Table 2).

## Discussion

In this study to elicit opinions about frailty in ESRD patients, there was consensus among clinicians who care for this population, and moderate consensus among surveyed adults with ESRD, that ESRD patients are more likely to be frail than healthy adults. There was some discordance between self-identification and measurement of frailty status, and participants who identified as frail were more likely to think that adults with ESRD are more likely to be frail, regardless of their own measured frailty status.

Four of the five Fried frailty components (exhaustion, low activity, slow walking speed and grip strength) were thought to be relevant to adults with ESRD, and clinicians were more likely to believe those four components were relevant. There was only moderate consensus among both clinicians and patients that unintentional weight loss characterizes frailty in this population, likely given the weight fluctuations and fluid shifts that are common in ESRD patients. Other possible ESRD-specific frailty components that were identified included history of falls, physical decline, and poor cognitive impairment. Interestingly, the novel components identified are less likely to be measures of kidney disease, but rather are downstream effects of ESRD. Finally, there was consensus among the clinicians that intradialytic foot peddlers and prehabilitation prior to KT would be effective interventions for reducing the burden of frailty in this vulnerable population.

Prior research has shown that frailty, as measured by the Fried phenotype, is an important predictor of ESRD and transplant patient outcomes [5–13]. A prior study has identified 67 different instruments for the study of frailty among older adults [20]; however, none of these instruments are specific to patients with ESRD. Our findings suggest that physicians and patients believe that exhaustion, low physical activity, slow walking speed, and poor grip strength were relevant to ESRD patients but that weight loss was possibly not relevant.

Interventions such as intradialytic activities and prehabilitation have been proposed to improve frailty among adults with ESRD [21, 22]. Our findings are consistent with a previous qualitative study of hemodialysis patients that reported patient support for intradialytic exercise [21]. We extended these findings to include ESRD patient and clinician opinions on whether intradialytic exercise using a foot peddler would improve physiologic reserve. Additionally, we included clinician and patient opinions on prehabilitation prior to transplantation; there was consensus among both groups that prehabilitation would help ESRD patients and make them less frail. This perception is consistent with studies that demonstrate that frailty is associated with physical functioning in older adults [1].

Clinicians had higher levels of consensus on all issues relating to frailty in ESRD than did patients. Given the discordance between frailty status and self-perception of frailty found in this study, the lower levels of consensus among patients might be related to a lack of awareness of frailty and its prevalence in ESRD.

A strength of this study was the two round Delphi study design of clinicians caring for ESRD patients, which uses both qualitative and quantitative methods to elicit opinion and facilitate consensus. Additionally, this is the first study to directly survey ESRD patients on their opinions regarding what makes them frail. This study’s main limitation was the single-center design with respect to sampling patients and clinicians at Johns Hopkins. Perspectives of clinicians and patients from one center may not be generalizable. The wording of the survey items, particularly those questions relating to clinician perceptions of acceptability of interventions to improve frailty, may have elicited an affirmative response.

Nephrologists, transplant surgeons and geriatricians may all have incomplete perspectives on frailty in ESRD given the limited populations with whom they work. However, all clinician participants had experience treating adults with ESRD, as this was an inclusion criterion for this study. Despite these limitations, 85% of clinicians who treat adults with ESRD at Johns Hopkins were represented in the study, and the sample size was consistent with or larger than prior Delphi studies [17, 23].

As the target population for this study was ESRD patients who were pursuing kidney transplantation, patients included in this study were all under evaluation for kidney transplantation. With an 80.6% response rate, we believe this study is representative of ESRD patients pursuing kidney transplantation at our center. Patients being evaluated for transplantation might be concerned that their appearing frail would affect their listing for transplantation, which could bias their responses. However, patients were assured that none of their responses would affect any aspect of their care during evaluation, including their access to transplantation. Furthermore, the questions in this survey are part of a longer 45-min assessment consisting of 252 questions. Thus the likelihood of responses being biased by a desire to appear not frail should be mitigated. Additionally, ESRD patients were not asked to suggest potential novel ESRD-specific frailty components.

The Fried frailty measure is just one of many measures that could be used in an ESRD setting. However, in a recent review of 67 different frailty assessment instruments, the Fried phenotype was the most cited instrument [20]. The Fried phenotype is commonly used in ESRD research [24–26] and its biological basis is well established [27]. Given its prevalence, particularly in ESRD research, we sought to assess its appropriateness in an ESRD setting.

This study is one step in the process of refining the definition of frailty in ESRD. This process should also include structured reviews and qualitative studies to elicit further themes relating to frailty in ESRD. Any new measure of frailty in ESRD patients should be thoroughly validated before informing clinical decisions. Likewise, any new components added to this measure should be further studied and operationalized prior to its inclusion in a definition of frailty.

In a given population, the clinical usefulness of a definition of frailty depends on the relevance of its components to the physiology of that population. Among ESRD patients and clinicians who care for them, there is consensus that many Fried frailty components measure decline of physiologic reserve in ESRD patients; however, the complex physiologic issues experienced by patients with ESRD may make other aspects of physiologic reserve, like history of falls or physical decline, relevant in this population. An ESRD-specific measure of frailty could improve risk prediction in ESRD and transplant patients, and the patient and clinician opinions elicited in this study should inform the development of such a measure.

## Conclusions

Clinicians and patients agreed that frailty is more common among adults with ESRD than the general population. Unintentional weight loss was seen as less relevant to characterizing frailty in adults with ESRD, but clinicians identified history of falls, physical decline, and cognitive impairment as possible new components of frailty in this population. Clinicians and patients agreed that intradialytic foot peddlers would make ESRD patients less frail. These findings contribute to the refining of the frailty phenotype for ESRD patients, and to the identification of interventions to improve physiologic reserve in this population.

## Additional files

Additional file 1: Table S1. Delphi Study Surveys of Clinicians who treat adults with ESRD: First Survey. (DOCX 86 kb)

Additional file 2: Table S2. Validated Fried Frailty Assessment Tool. (DOCX 20 kb)

Additional file 3: Table S3. Delphi Study Survey of Clinicians who treat adults with ESRD: Second Survey. (DOCX 74 kb)

Additional file 4: Table S4. Description of data: Survey of Adults with ESRD undergoing hemodialysis. (DOCX 83 kb)

## Abbreviations

ESRD: End stage renal disease
KT: Kidney transplantation
SD: Standard deviation
