# Supplementary material for: Engaging clinicians and patients to assess and improve frailty measurement in adults with end stage renal disease
Source: BMC Nephrol. 2018 Jan 12;19:8. doi: 10.1186/s12882-017-0806-0 (PMC5766981; doi:10.1186/s12882-017-0806-0)
Supplement: Supplementary file 1 — Delphi Study Surveys of Clinicians who treat adults with ESRD: First Survey. (DOCX 86 kb) [file 12882_2017_806_MOESM1_ESM.docx]

Supplemental Table 1: Delphi Study Surveys of Clinicians who treat adults with ESRD: First Survey

| Item | Response Option |
| --- | --- |
| *Frailty* | |
| Do you think adults with ESRD are more likely or less likely to be frail than healthy adults? | - More likely - Less likely - About the same |
| The frailty phenotype is comprised of 5 components and the presence of 3 or more signifies frailty. Are the listed components relevant to patients with ESRD? | - Relevant to patients with ESRD - Not relevant to patients with ESRD and should be removed*   *Clinicians who responded “Not relevant” were prompted: “Please briefly explain why you would remove [component]” |
| Are there additional components that characterize frailty in adults with ESRD that you would add to the list? | - Yes - No   *Clinicians who responded “Yes” were prompted: “Please list the components that you would add” |
| *Interventions* | |
| Do you think that patients with ESRD could improve physical function by using a foot peddler while undergoing dialysis? A foot peddler is like a bike that you could use while seated in the dialysis chair. | - Yes - No - Don’t know |
| Do you think using a foot peddler would make a person undergoing dialysis less frail? |  |
| Do you think your patients would be interested in using a foot peddler while on dialysis? |  |
| Do you think prehabilitation prior to transplantation could help improve post-operative outcomes in adults with ESRD? |  |
| Do you think prehabilitation could make someone undergoing a kidney transplant less frail? |  |
| Do you think your patients would be interested in prehabilitation before their kidney transplant? |  |
