# Supplementary material for: Engaging clinicians and patients to assess and improve frailty measurement in adults with end stage renal disease
Source: BMC Nephrol. 2018 Jan 12;19:8. doi: 10.1186/s12882-017-0806-0 (PMC5766981; doi:10.1186/s12882-017-0806-0)
Supplement: Supplementary file 2 — Validated Fried Frailty Assessment Tool. (DOCX 20 kb) [file 12882_2017_806_MOESM2_ESM.docx]

Supplemental Table 2**:** Validated Fried frailty assessment tool [1, 2]

| Component | Description |
| --- | --- |
| Unintentional weight loss | Based on patient’s self-reported current weight and weight 1 year ago. Patients reporting unintentional loss of ≥10lb (≥4.5 kg) over the past year were considered to have the weight loss component present. |
| Exhaustion | Based on responses to 2 questions from the CES-D scale [3]. Patients reporting either feeling “that everything I did was an effort” or “I could not get going” for ≥3 days in the past week were considered to have the exhaustion component present. |
| Physical activity | Frequency of physical activity was assessed with the short version of the Minnesota Leisure Time Activity questionnaire [4]. Kilocalories expended per week were calculated using a standardized algorithm (number of days physical activity took place in the past 2 weeks x duration of activity (minutes) x number of kilocalories expended per minute). Patients were considered to have the physical activity component present if they expended <383 kcal/week (men) or <270 kcal/week (women). |
| Walking Speed | Patients were timed while walking 15 ft (4.5 m); speeds were stratified by sex and height. The walking speed component was considered present in men who were ≤173 cm and required ≥7 s, men who were >173 cm and required 6 s, women who were ≤159 cm and required ≥7 s, and women who were >159 cm and required ≥6 s. |
| Grip Strength* | Measured using a Jamar handheld dynamometer (kg); stratified by sex and BMI. Cutoffs for presence of grip strength component were: |
| Men | |
| BMI ≤24.0 | ≤29 |
| BMI = 24.1-28.0 | ≤30 |
| BMI > 28.0 | ≤32 |
| Women | |
| BMI ≤23.0 | ≤17 |
| BMI = 23.1-26.0 | ≤17.3 |
| BMI = 26.1-29.0 | ≤18 |
| BMI > 29.0 | ≤21 |

1. Fried LP, Tangen CM, Walston J, Newman AB, Hirsch C, Gottdiener J, Seeman T, Tracy R, Kop WJ, Burke G *et al*: **Frailty in older adults: evidence for a phenotype**. *J Gerontol A Biol Sci Med Sci* 2001, **56**(3):M146-156.

2. Garonzik-Wang JM, Govindan P, Grinnan JW, Liu M, Ali HM, Chakraborty A, Jain V, Ros RL, James NT, Kucirka LM *et al*: **Frailty and delayed graft function in kidney transplant recipients**. *Archives of surgery (Chicago, Ill : 1960)* 2012, **147**(2):190-193.

3. Radloff LS: **The CES-D Scale: A Self-Report Depression Scale for Research in the General Population**. *Applied Psychological Measurement* 1977, **1**(3):385-401.

4. Taylor HL, Jacobs DR, Schucker B, Knudsen J, Leon AS, Debacker G: **A questionnaire for the assessment of leisure time physical activities**. *Journal of Chronic Diseases* 1978, **31**(12):741-755.
