# Supplementary material for: Engaging clinicians and patients to assess and improve frailty measurement in adults with end stage renal disease
Source: BMC Nephrol. 2018 Jan 12;19:8. doi: 10.1186/s12882-017-0806-0 (PMC5766981; doi:10.1186/s12882-017-0806-0)
Supplement: Supplementary file 3 — Delphi Study Survey of Clinicians who treat adults with ESRD: Second Survey. (DOCX 74 kb) [file 12882_2017_806_MOESM3_ESM.docx]

Supplemental Table 3: Delphi Study Survey of Clinicians who treat adults with ESRD: Second Survey

| Survey Item | Response Options |
| --- | --- |
| *Frailty* | |
| 20% of clinicians surveyed believe that unintentional weight loss is not relevant to adults with ESRD because weight often fluctuates in this population. Should "unintentional weight loss" be removed from the frailty phenotype for adults with ESRD? | - Yes - No |
| 8% of clinicians surveyed believe that slowed walking is not relevant to adults with ESRD. Should "slowed walking" be removed from the frailty phenotype for adults with ESRD? |  |
| 2.5% of clinicians surveyed believe that weak strength is not relevant to adults with ESRD. Should "weak strength" be removed from the frailty phenotype for adults with ESRD? |  |
| 24% of clinicians surveyed believe there are additional components that characterize frailty in adults with ESRD that should be added to the list. The following were suggested as aspects of ESRD that contribute to the loss of physiologic reserve in adults with ESRD. Please check all of the components that you think should be added to the frailty phenotype for adults with ESRD. | - Cognition - Poor nutrition or diet - Albumin - Health care utilization - Metabolic bone disease - History of falls - Physical decline - Excess fluid - Ultrafiltration - Other (Please describe) |
