# Supplementary material for: Engaging clinicians and patients to assess and improve frailty measurement in adults with end stage renal disease
Source: BMC Nephrol. 2018 Jan 12;19:8. doi: 10.1186/s12882-017-0806-0 (PMC5766981; doi:10.1186/s12882-017-0806-0)
Supplement: Supplementary file 4 — Description of data: Survey of Adults with ESRD undergoing hemodialysis. (DOCX 83 kb) [file 12882_2017_806_MOESM4_ESM.docx]

Supplemental Table 4: Survey of Adults with ESRD undergoing hemodialysis

| Item | Response Options |
| --- | --- |
| *Frailty** | |
| Do you think that patients with ESRD are more or less likely to be frail than other people? | - More likely - Less likely - About the same |
| Which of the following best describes you? | - Not frail - Somewhat frail - Frail - Don’t know |
| Do you think the following things make a person frail?   - Unintentional weight loss - Slowed walking - Low physical activity - Exhaustion - Poor memory | - Yes - No |
| Has a doctor ever told you that you are frail? |  |
| Do you want to be told by a doctor whether or not you are frail? |  |
| *Interventions* | |
| A foot peddler is like a bike that you could use while seated in the dialysis chair. Would you want to use a foot peddler while on dialysis? | - Yes - No |
| Do you think that using a foot peddler would make a person undergoing dialysis less frail overall? |  |
| Do you think that prehabilitation could help people with ESRD before they get a transplant? |  |
| Do you think that prehabilitation would make a person getting a kidney transplant less frail? |  |
| Would you be interested in being part of a prehabilitation program up until your kidney transplant? |  |

*The survey included the following language to describe the term “frailty:” “Frailty is a syndrome characterized by a loss of physiologic reserve. People who are frail are unable to bounce back after they get sick or hurt”
